# Differential Effects of Dupilumab in a Patient With Localized Heat Urticaria, Delayed Solar Urticaria, and Symptomatic Dermographism

**DOI:** 10.1111/1346-8138.70423

**Published:** 2026-07-23

**Authors:** Shuta Yoshitani, Shinichi Moriwaki, Atsushi Fukunaga

**Affiliations:** ^1^ Department of Dermatology, Division of Medicine for Function and Morphology of Sensory Organs, Faculty of Medicine Osaka Medical and Pharmaceutical University Takatsuki Osaka Japan

**Keywords:** chronic inducible urticaria, dupilumab, omalizumab, solar urticaria, symptomatic dermographism

## Abstract

Physical urticaria is a subtype of chronic inducible urticaria characterized by wheals and/or angioedema triggered by physical stimuli. We report a refractory case of combined localized heat urticaria, delayed solar urticaria, and symptomatic dermographism in a 16‐year‐old girl. Provocation testing showed erythematous and edematous lesions after thermal stimulation at 42°C and after the application of warm water at 40°C and 42°C. An intradermal test using the patient's serum heated to 42°C for 5 min yielded a positive result, leading to the diagnosis of localized heat urticaria. Visible light irradiation induced no immediate wheals; however, wheals developed at all irradiated sites 4.5 h later, accompanied by intense pruritus without burning sensation or pain, consistent with delayed solar urticaria. A FricTest provoked wheals, confirming symptomatic dermographism. Antihistamines were ineffective, and omalizumab, administered as a second‐line treatment, improved only the symptomatic dermographism, with no effect on the heat or solar urticaria. Dupilumab was subsequently administered 12 times. Repeated provocation testing during treatment showed resolution of symptomatic dermographism, whereas localized heat urticaria persisted and delayed solar urticaria showed no meaningful improvement. The Urticaria Control Test score also did not improve. These findings suggest that the pathogenesis of symptomatic dermographism in this case may involve IgE‐ and IL‐4/IL‐13‐mediated pathways, whereas localized heat urticaria and delayed solar urticaria may be driven by distinct mechanisms.

## 1 Introduction

Physical urticaria is a type of chronic inducible urticaria characterized by the appearance of wheals or angioedema in response to physical stimuli [1]. Dupilumab has been reported to be effective in treating chronic spontaneous urticaria (CSU) [2], but there are few reports of its efficacy in physical urticaria. We report a refractory case of combined localized heat urticaria, delayed solar urticaria, and symptomatic dermographism in which dupilumab produced differential effects depending on the urticaria subtype.

## 2 Case Report

The patient was a 16‐year‐old girl with a history of asthma. Since childhood, she had experienced urticaria during bathing, at sun‐exposed sites several hours after sun exposure, and at sites of mechanical stimulation. All types of urticaria were accompanied by itchiness.

Blood tests showed no abnormalities, with an eosinophil count of 50/μL, a total IgE concentration of 145 IU/mL (within the reference range of < 173 IU/mL), blood protoporphyrin of 47 μg/dL red blood cells (RBC) (reference range, 30–86 μg/dL RBC), blood coproporphyrin of ≤ 1 μg/dL RBC (reference range, ≤ 1 μg/dL RBC), and urinary coproporphyrin of 50 μg/g creatinine (reference range, ≤ 170 μg/g creatinine).

A thermal stimulation test was performed on the lower extremities at 42°C for 5 min. This resulted in the appearance of erythematous and edematous lesions at the exposed sites 10 min after stimulation (Figure 1a), and the patient reported feeling unwell. When a beaker filled with warm water was applied to the abdomen, no erythema was observed at 21°C or 38°C, whereas pruritic edematous erythema and wheals appeared at 40°C and 42°C 10 min after stimulation (Figure 1b). An intradermal test using the patient's serum heated to 42°C for 5 min yielded a positive result, confirming that the serum contained a urticaria‐inducing factor (Figure 1c). Thus, she was diagnosed with localized heat urticaria associated with a serum urticaria‐inducing factor.

**FIGURE 1:**
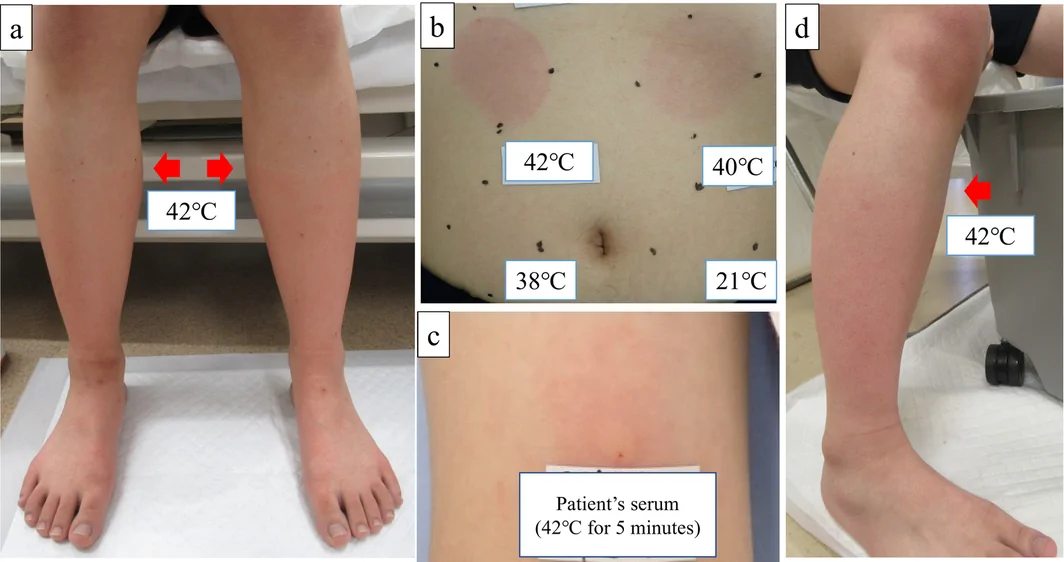
Provocation tests (heat urticaria) (a–c) before biologic treatment and (d) during dupilumab therapy. (a) A thermal stimulation test was performed on the lower extremities by immersion in 42°C water for 5 min, inducing erythematous and edematous lesions; the red arrows indicate the water level (height of stimulation). (b) During a thermal stimulation test performed on the abdomen, no erythema was observed at 21°C or 38°C, whereas pruritic edematous erythema and wheals appeared at 40°C and 42°C 10 min after stimulation. (c) An intradermal test using the patient's serum heated to 42°C for 5 min yielded a positive result. (d) A thermal stimulation test during dupilumab therapy again induced erythematous and edematous lesions; the red arrows indicate the water level, demonstrating no improvement.

Her back was irradiated with visible light at a distance of 30 cm for 1, 5, 10, and 15 min. No wheals were observed immediately after irradiation (Figure 2a); however, wheals developed at all sites after 4.5 h, irrespective of the duration of irradiation (Figure 2b). The wheals were associated with intense pruritus but not with a burning sensation or pain. They resolved within a few hours without residual pigmentation (Figure 2c). An intradermal test with serum irradiated with visible light for 10 min yielded no wheals (Figure 2d). Irradiation with ultraviolet A and ultraviolet B light did not induce urticarial lesions. The minimal erythema dose was 120 mJ/cm^2^, and ultraviolet A irradiation at 10 J/cm^2^ did not induce erythema, indicating no reduction in the minimal erythema dose or minimal reaction dose. Solar urticaria develops within 5–15 min in 75%–95% of cases, although delays of more than 1 h have been reported [3, 4]. By contrast, porphyria may be associated with a burning sensation and pain rather than pruritus [5]. In this case, blood and urinary porphyrin levels were within the reference ranges; therefore, porphyria was considered unlikely. Accordingly, the patient was diagnosed with delayed solar urticaria (action spectrum: visible light) with a negative serum factor.

**FIGURE 2:**
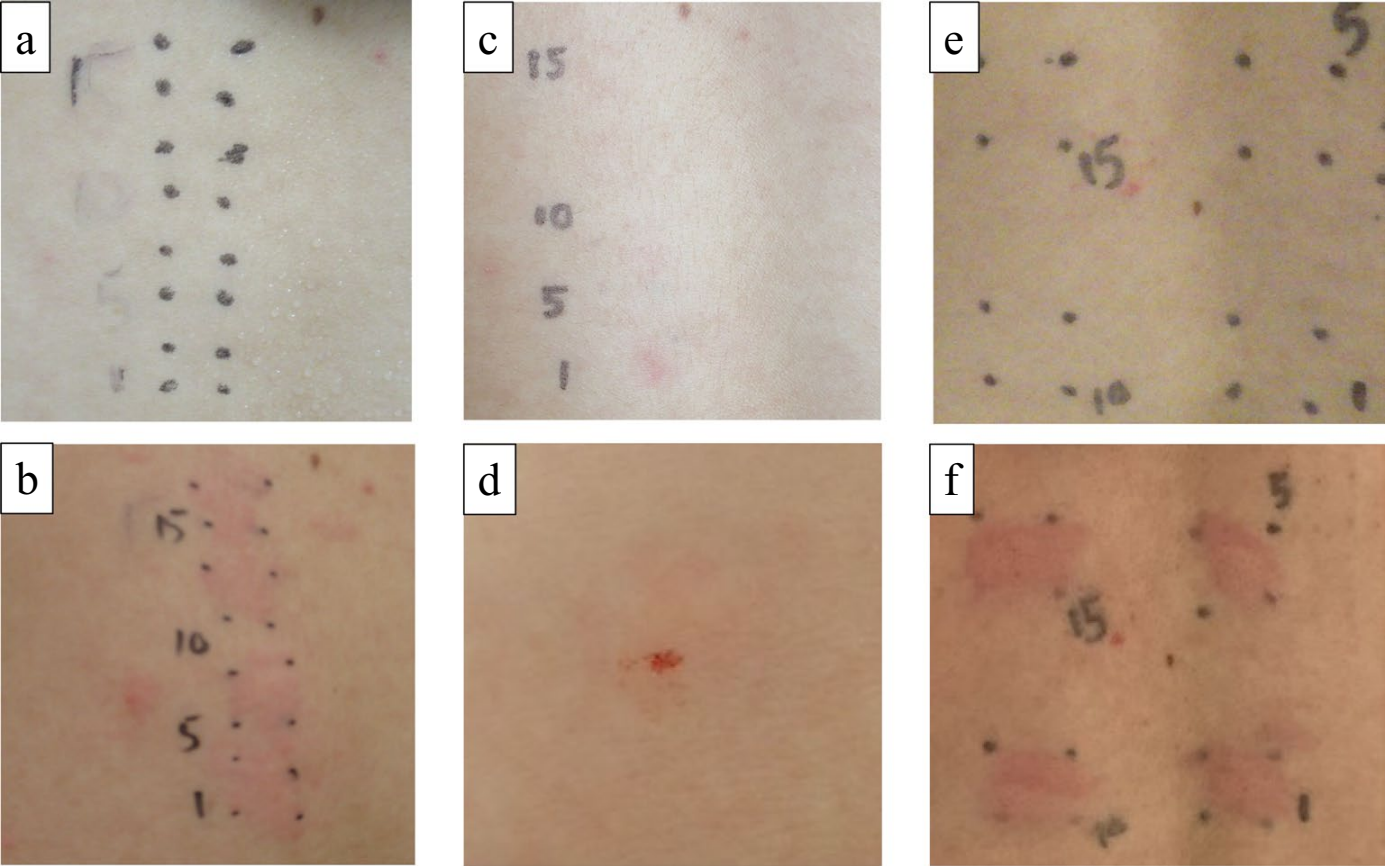
Provocation tests (solar urticaria) (a–d) before biologic treatment and (e, f) during dupilumab therapy. (a–c) A visible light irradiation test using a slide projector lamp with a 250‐W xenon bulb (VIP300 M/Z; Yokogawa Electric Co., Japan) was performed on the back at a distance of 30 cm for 1, 5, 10, and 15 min. (a) No wheals were observed immediately after irradiation. However, (b) wheals developed 4.5 h later and (c) had resolved by the following day. (d) An intradermal test with serum irradiated with visible light for 10 min yielded no wheals. (e) A visible light irradiation test performed on the back did not induce wheals 4.5 h after irradiation. (f) However, wheals appeared at all test sites 7 h after exposure.

Following stroking with the FricTest device, a wheal was observed at the site of stimulation (Figure 3a). Therefore, the patient was diagnosed with symptomatic dermographism.

**FIGURE 3:**
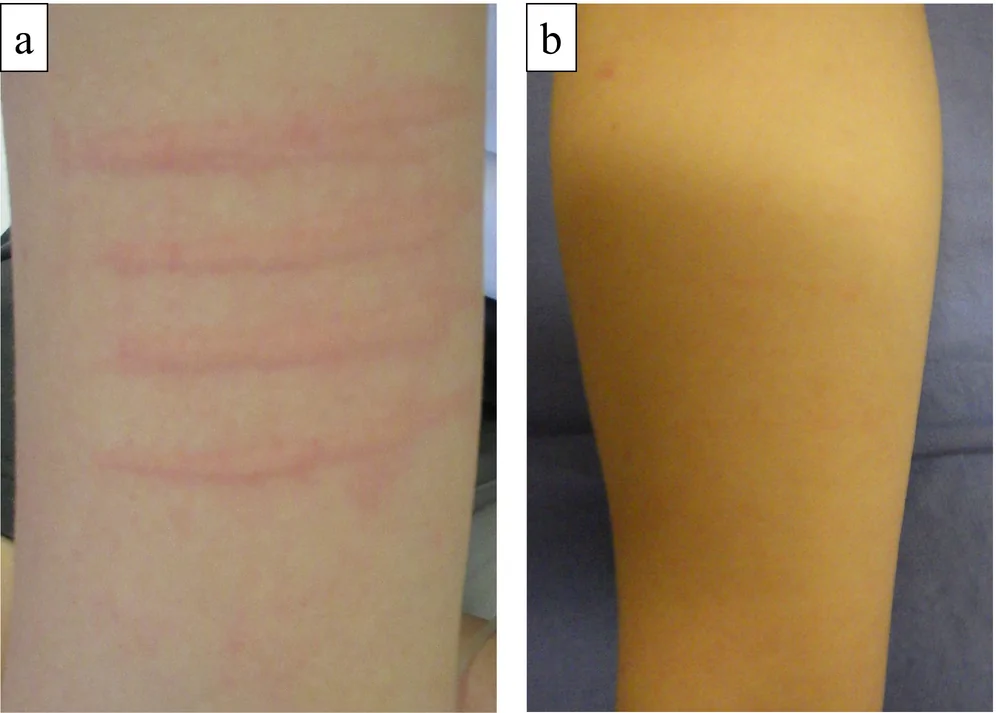
Provocation tests (symptomatic dermographism) (a) before biologic treatment and (b) during dupilumab therapy. (a) A FricTest performed on the upper arm induced a wheal at the site of stimulation within 10 min. (b) A FricTest performed on the upper arm did not induce wheals, indicating improvement of symptomatic dermographism.

These provocation tests were performed in the absence of any concomitant medication.

Urticarial episodes also occurred in the absence of identifiable eliciting factors, leading to a diagnosis of CSU. Despite treatment with oral antihistamines (bilastine, rupatadine fumarate) and an H2 antagonist (lafutidine), the Urticaria Control Test (UCT) score was 3. As a second‐line treatment, omalizumab was administered six times. Although the symptomatic dermographism improved, there was no marked response in the heat or solar urticaria, and the UCT score remained 6 (the minimal important difference is 3 points). Consequently, dupilumab was administered because of the failure of existing treatments.

Dupilumab was administered a total of 12 times, and provocation testing was performed while the patient was receiving dupilumab alone, without concomitant antihistamine treatment. A thermal stimulation test resulted in the appearance of erythematous and edematous lesions (Figure 1d). Irradiation with visible light did not induce urticarial lesions within 4.5 h (Figure 2e), but wheals were observed at all irradiated sites after 7 h (Figure 2f). Although the induction time of solar urticaria was slightly prolonged after dupilumab treatment, subjective symptoms remained unchanged, and the timing of solar urticaria onset after irradiation had fluctuated from day to day even before treatment, indicating limited efficacy. A FricTest did not induce any wheals (Figure 3b). Blood tests revealed an eosinophil count of 76/μL and a total IgE concentration of 115 IU/mL, accompanied by a slight reduction in IgE levels. Following dupilumab administration, the UCT score remained at 4, indicating no improvement. In summary, these findings suggest that dupilumab was effective in the treatment of symptomatic dermographism but ineffective in the management of heat urticaria and solar urticaria. Furthermore, because the UCT score did not improve, dupilumab was discontinued. No improvement in pruritus was observed with either omalizumab or dupilumab.

## 3 Discussion

The pathogenesis of physical urticaria is thought to be primarily mediated by type I (IgE‐mediated) allergy [1, 6]. In some cases, autoantigens produced by physical or environmental triggers stimulate mast cells via IgE. This process leads to mast cell activation and degranulation, resulting in the release of histamine and other inflammatory mediators [7, 8]. Nevertheless, the triggering factors remain poorly understood. Second‐generation H1 antihistamines are the first‐line therapy, and omalizumab is recommended as an effective second‐line treatment for physical urticaria refractory to antihistamines [3]. Recent research has demonstrated the efficacy of the IL‐4/IL‐13 receptor monoclonal antibody dupilumab in treating CSU [2]. Dupilumab may suppress an upstream pathogenic mechanism by inhibiting IL‐4/IL‐13‐mediated IgE production by B cells. Moreover, IL‐4 and IL‐13 can contribute to direct mast cell activation, immune cell trafficking to the skin, and neuronal sensitization, resulting in pruritus [9, 10]. Dupilumab may also improve physical urticaria by inhibiting these IgE‐independent pathways. However, only a limited number of reports have suggested that dupilumab is effective for physical urticaria [11, 12, 13].

In the present case, both omalizumab and dupilumab improved the symptomatic dermographism. However, neither omalizumab nor dupilumab improved the localized heat urticaria or delayed solar urticaria. These findings suggest that the pathogenesis of symptomatic dermographism in this case was mediated, at least in part, by IgE and IL‐4/IL‐13. By contrast, the lack of efficacy of both omalizumab and dupilumab against the localized heat urticaria and delayed solar urticaria suggests that factors other than IgE and IL‐4/IL‐13 were involved in the pathogenesis of these conditions. CSU and chronic inducible urticaria sometimes coexist in the same patient. Although they share common features, they are generally considered distinct entities with different underlying causes and responses to treatment. However, a previous report described a patient with CSU, pressure urticaria, and symptomatic dermographism in whom all three conditions showed complete remission after omalizumab treatment [14], and CSU, symptomatic dermographism, cholinergic urticaria, and cold urticaria are known to frequently coexist [15]. Therefore, we speculate that the pathogenesis of CSU and symptomatic dermographism may involve similar mechanisms, including IgE and IL‐4/IL‐13. In contrast, rare forms of physical urticaria, such as heat urticaria and delayed solar urticaria, may involve pathogenic mechanisms distinct from those of CSU. Further accumulation of cases and elucidation of the pathogenesis of physical urticaria are needed.

## Ethics Statement

The authors have nothing to report.

## Consent

The patient and her guardians provided informed consent for the publication of this report.

## Conflicts of Interest

A.F. has received research funding from Taiho Pharmaceutical Co. Ltd. and lecture fees from Novartis Pharma K.K., Sanofi K.K., Kyowa Kirin Co. Ltd., Kyorin Pharmaceutical Co. Ltd., Mitsubishi Tanabe Pharma Corporation, and Taiho Pharmaceutical Co. Ltd., outside the submitted work. S.M. is an Editorial Board member of *The Journal of Dermatology* and a co‐author of this article. To minimize bias, they were excluded from all editorial decision‐making related to the acceptance of this article for publication.

## Data Availability

The data that support the findings of this study are available on request from the corresponding author. The data are not publicly available due to privacy or ethical restrictions.
